# Qili Qiangxin capsule as adjunctive therapy for heart failure after acute myocardial infarction: a GRADE-assessed systematic review and meta-analysis of randomised controlled trials

**DOI:** 10.3389/fphar.2026.1959187

**Published:** 2026-10-19

**Authors:** Jun-Hong Yu, Hao-Yu Yu, Jun-Cheng Zhang, Zhao-Yang Chen, Cong Wang, You-Qi Wu, Yi-Fei Wang, Nicola Robinson, Jian-Ping Liu, Yun-Lun Li, Shi-Bing Liang

**Affiliations:** 1 Centre for Evidence-Based Chinese Medicine, Beijing University of Chinese Medicine, Beijing, China; 2 School of Medicine, Shandong University of Traditional Chinese Medicine, Jinan, China; 3 Cardiovascular Disease Center, Affiliated Hospital of Shandong University of Traditional Chinese Medicine, Jinan, China; 4 Science and Technology Department, Affiliated Hospital of Shandong University of Traditional Chinese Medicine, Jinan, China; 5 Institute of Health and Social Care, London South Bank University, London, United Kingdom; 6 Centre for Evidence-Based Medicine, Hospital of Chengdu University of Traditional Chinese Medicine, Chengdu, China; 7 Sichuan Provincial Centre for Evidence-Based Chinese Medicine, Hospital of Chengdu University of Traditional Chinese Medicine, Chengdu, China; 8 Clinical Study Center, Affiliated Hospital of Shandong University of Traditional Chinese Medicine, Jinan, China; 9 Centre for Evidence-Based Chinese Medicine, Shandong University of Traditional Chinese Medicine, Jinan, China; 10 Shandong Big Data Research Association, Jinan, China

**Keywords:** heart failure, meta-analysis, myocardial infarction, Qili Qiangxin capsule, systematic review

## Abstract

**Background:**

Heart failure (HF) after acute myocardial infarction (AMI) remains a major cause of morbidity and mortality despite advances in conventional therapy. Qili Qiangxin Capsule (QLQX) has been widely used as an adjunctive treatment for HF in China, but its efficacy and safety in patients with HF after AMI require further evaluation.

**Methods:**

Seven databases were searched from their inception to January 2026. Randomised controlled trials (RCTs) comparing QLQX plus conventional Western medicine (WM) with WM alone were included. The primary outcome was N-terminal pro-B-type natriuretic peptide (NT-proBNP). Secondary outcomes included left ventricular ejection fraction (LVEF), B-type natriuretic peptide (BNP), left ventricular end-diastolic diameter (LVEDD), left ventricular end-systolic diameter (LVESD), left ventricular end-diastolic volume (LVEDV), clinical response rate, high-sensitivity C-reactive protein (hs-CRP), quality of life, all-cause mortality, heart failure-related hospitalisation, and adverse events. Meta-analysis was performed using RevMan 5.4.

**Results:**

A total of 16 RCTs involving 1391 participants were included. Compared with WM alone, QLQX plus WM was associated with greater reductions in NT-proBNP, BNP, LVEDD, LVESD, and hs-CRP, and improving LVEF, quality of life and the clinical response rate. No statistical differences were observed in LVEDV, all-cause mortality, and adverse events. Heart failure-related hospitalisation was not reported in any included study. The certainty of evidence for all outcomes was rated as low or very low according to the GRADE approach.

**Conclusion:**

QLQX plus conventional WM was associated with improvements in several surrogate and short-term clinical outcomes in patients with HF after AMI. However, the certainty of evidence was low or very low, and data on mortality and heart failure-related hospitalisation were insufficient. The current evidence is therefore inadequate to determine whether QLQX improves long-term clinical prognosis. Further high-quality RCTs with longer follow-up and clinically important endpoints are needed.

**Systematic Review Registration:**

https://www.crd.york.ac.uk/PROSPERO/view/CRD420261277047, identifier CRD420261277047.

## 1 Introduction

Heart failure (HF) is a major end-stage manifestation of cardiovascular diseases and is associated with high rates of disability and mortality. With the acceleration of global population aging, the prevalence of HF continues to increase worldwide, posing a substantial public health burden (Ambrosy et al., 2014). Acute myocardial infarction (AMI), a major manifestation of coronary artery disease, is one of the leading causes of HF. Epidemiological studies have shown that approximately 25% of patients with ST-segment elevation myocardial infarction develop HF, and the rehospitalization rate due to HF after myocardial infarction reaches 16.7% (Li et al., 2019; Chen et al., 2022). Despite continuous advances in pharmacological and non-pharmacological therapies, the risks of all-cause mortality, rehospitalization, and cardiovascular events in these patients remain unacceptably high (Han et al., 2015; Li et al., 2015; Roger, 2021).

Current management of HF after AMI mainly includes guideline-directed medical therapy, coronary revascularization procedures, and device or surgery based interventions (Branch of Cardiovascular Physicians et al., 2020; Heidenreich et al., 2022). Although these interventions have improved clinical outcomes to some extent, the risks of ventricular remodeling, recurrent hospitalization, and adverse cardiovascular events remain high (Frantz et al., 2022). In addition, long-term multidrug therapy may increase the risk of adverse reactions and poor treatment adherence (Lee et al., 2026). Therefore, safe and effective adjunctive therapies are needed to further improve outcomes in patients with HF after AMI.

Traditional Chinese medicine (TCM) has increasingly been used as a complementary therapy for cardiovascular diseases in China (Jia et al., 2020). Qili Qiangxin Capsule (QLQX), a Chinese patent medicine, has been widely used as an adjunctive treatment for HF in China (Xing et al., 2022). Modern pharmacological studies have shown that QLQX may improve myocardial energy metabolism and inhibit ventricular remodeling (Wang, 2018; Zhong and Wang, 2024). Although a number of randomised controlled trials (RCTs) have investigated the efficacy of QLQX in patients with HF after AMI, no systematic review and meta-analysis has comprehensively evaluated its efficacy and safety in this specific population.

To address this gap, we conducted a systematic review and meta-analysis to comprehensively evaluate the efficacy and safety of QLQX as an adjunct to conventional Western medicine (WM) in patients with HF after AMI. In addition, the Grading of Recommendations Assessment, Development and Evaluation (GRADE) approach was applied to assess the certainty of the evidence and provide a more robust basis for clinical decision-making.

## 2 Methods

This review adhered to the Preferred Reporting Items for Systematic Reviews and Meta-Analyses (PRISMA) statement (Page et al., 2021). The PRISMA 2020 Checklist is provided in Supplementary Material 1. The research protocol was prospectively registered on PROSPERO (registration number: CRD420261277047).

### 2.1 Search strategy

A systematic literature search was conducted across seven databases, including China National Knowledge Infrastructure (CNKI), Wanfang Database, China Science and Technology Journal Database (VIP database), China Biomedical Literature Database (CBM), PubMed, the Cochrane Library, and Embase from their inception to January 2026. The publication type was restricted to RCTs regardless of language. For the literature search, MeSH terms included “Myocardial Infarction” and “Heart Failure”, while free-text terms included “Infarction, Myocardial”, “Cardiac Failure”, and “Qili Qiangxin”. Detailed search strategies for each database are provided in Supplementary Material 2.

### 2.2 Inclusion criteria

#### 2.2.1 Types of studies

Only RCTs were included.

#### 2.2.2 Types of participants

Patients diagnosed with AMI who subsequently developed HF judged by the original study authors to be secondary to the myocardial infarction were included, regardless of the timing of onset. There were no restrictions on age, disease duration, race, or geographic location.

#### 2.2.3 Types of interventions

The intervention group received QLQX in addition to WM. There were no restrictions on the dosage, formulation, or duration of QLQX treatment. The control group received either WM alone or WM plus a placebo matching QLQX. To ensure comparability, the WM regimen had to be identical in both groups. Studies with unclear treatment duration or substantial differences in treatment duration between groups were excluded.

#### 2.2.4 Types of outcomes

The primary outcome was N-terminal pro-B-type natriuretic peptide (NT-proBNP), as prespecified in the registered protocol. NT-proBNP was selected because it is a widely used and clinically relevant biomarker for evaluating disease severity and treatment response in patients with heart failure. The secondary outcomes were left ventricular ejection fraction (LVEF), B-type natriuretic peptide (BNP) in studies not reporting NT-proBNP, left ventricular end-diastolic diameter (LVEDD), left ventricular end-systolic diameter (LVESD), and left ventricular end-diastolic volume (LVEDV), clinical response rate (as defined by individual trials), high-sensitivity C-reactive protein (hs-CRP), quality of life (measured using validated scales, e.g., Minnesota Living with Heart Failure Questionnaire), all-cause mortality, heart failure-related hospitalisation, and adverse events, including serious adverse events.

### 2.3 Exclusion criteria

Studies lacking full text, containing incomplete data, or presenting significant errors were excluded.

### 2.4 Study selection and data extraction

All literature was managed using NoteExpress 4.1.0 software, with duplicate records removed prior to screening. The remaining studies were independently assessed for eligibility based on the predefined inclusion and exclusion criteria by two authors (JHY and HYY). Any disagreements were resolved through discussion, with arbitration by a third author (SBL). A flow diagram was created to illustrate the search results, including the number of included and excluded studies. For studies published multiple times, only the most comprehensive and up-to-date data were incorporated.

For all included RCTs, data extraction was performed independently by two authors (JHY and HYY) using a predefined form in Excel (2021). Disagreements during this process were resolved through discussion, with the involvement of a third author (SBL). Data extracted from each RCT included study title, author details, participant characteristics (age, sex, duration of illness, number randomised, number analysed), intervention and comparator details (dosage, frequency, duration), outcomes, and methodological elements relevant to risk of bias. For continuous outcomes, post-treatment values measured at the end of the respective treatment period were extracted. Although change-from-baseline values were prespecified for the primary outcome in the registered protocol, none of the included studies report change-from-baseline data with corresponding measures of variance. Therefore, end-of-treatment values were used for the meta-analysis. When multiple post-treatment assessment time points were reported, the measurement obtained at the end of the prespecified treatment period was preferentially selected. For dichotomous outcomes, the number of participants experiencing the event and the total number of participants in each group were extracted.

### 2.5 Trial quality assessment

Two authors (JHY and JCZ) independently assessed the risk of bias using the Cochrane risk of bias tool 2.0 (RoB 2.0) (Sterne et al., 2019). This tool evaluates five domains: the randomisation process, deviations from intended interventions, missing outcome data, measurement of the outcome, and selection of the reported result. Each domain was categorized as “low risk”, “some concerns”, or “high risk” of bias according to the tool’s criteria. Disagreements were resolved through discussion and reconciled by a third author (SBL).

### 2.6 Data synthesis

Statistical analyses were performed using RevMan 5.4 and STATA 17.0. For dichotomous outcomes, effect sizes were expressed as risk ratios (RRs) with 95% confidence intervals (CIs). For continuous outcomes, mean differences (MDs) were calculated when the same measurement scale and units were used across studies. Standardised mean differences (SMDs) were used when the same outcome was assessed using different measurement scales or units. Effect estimates were reported with 95% confidence intervals (CIs). Continuous outcomes were analysed using post-treatment values measured at the end of the respective treatment period. In the meta-analysis, a fixed-effect model was applied when heterogeneity was low (*I* ^2^ ≤ 50%). If substantial heterogeneity was observed (*I* ^2^ > 50%), data extraction and coding were rechecked for accuracy. In the absence of identifiable errors, a random-effects model was used to pool estimates, and findings were interpreted with caution. Subgroup analyses were planned to explore potential sources of heterogeneity considering factors such as timing of heart failure onset, treatment duration, and type of control intervention (placebo *versus* standard care alone), where sufficient and comparable information was available. Sensitivity analyses were performed by sequentially excluding individual studies for the primary outcome and by excluding studies judged to have an overall high risk of bias for outcomes to which such studies contributed data, to assess the robustness of the pooled estimates. If a sufficient number of trials were identified (>10), funnel plots were generated to assess the risk of publication bias.

### 2.7 GRADE certainty assessment of the evidence

The evidence certainty was assessed using the GRADE approach (Guyatt et al., 2008). The certainty of evidence for each outcome was evaluated as high, moderate, low, or very low according to the following five GRADE domains: risk of bias, inconsistency, indirectness, imprecision, and publication bias. The GRADE assessment was independently conducted by two reviewers (JHY and ZYC), and discrepancies were resolved through discussion with a third reviewer (SBL).

## 3 Results

### 3.1 Search results

As shown in Figure 1, a total of 310 studies were initially retrieved from the seven databases searched. After removing duplicates, 155 studies remained. Following title and abstract screening, 126 studies were excluded, leaving 29 studies for full-text assessment. One study could not be obtained in full text. After reviewing the full texts, 12 studies were excluded. Ultimately, 16 RCTs were included in the review (Hou, 2010; Xi et al., 2011; Liu, 2012; Bai et al., 2013; Pang et al., 2014; Wang et al., 2015; Qiao, 2018; Chen, 2019; Luo, 2019; Chen et al., 2020a; Xie et al., 2022; Liu et al., 2023; Han et al., 2024; Qi et al., 2024; Shi, 2024; Chen, 2025). The list of studies excluded at the full-text review stage, along with the reasons for exclusion, is provided in Supplementary Material 3.

**FIGURE 1:**
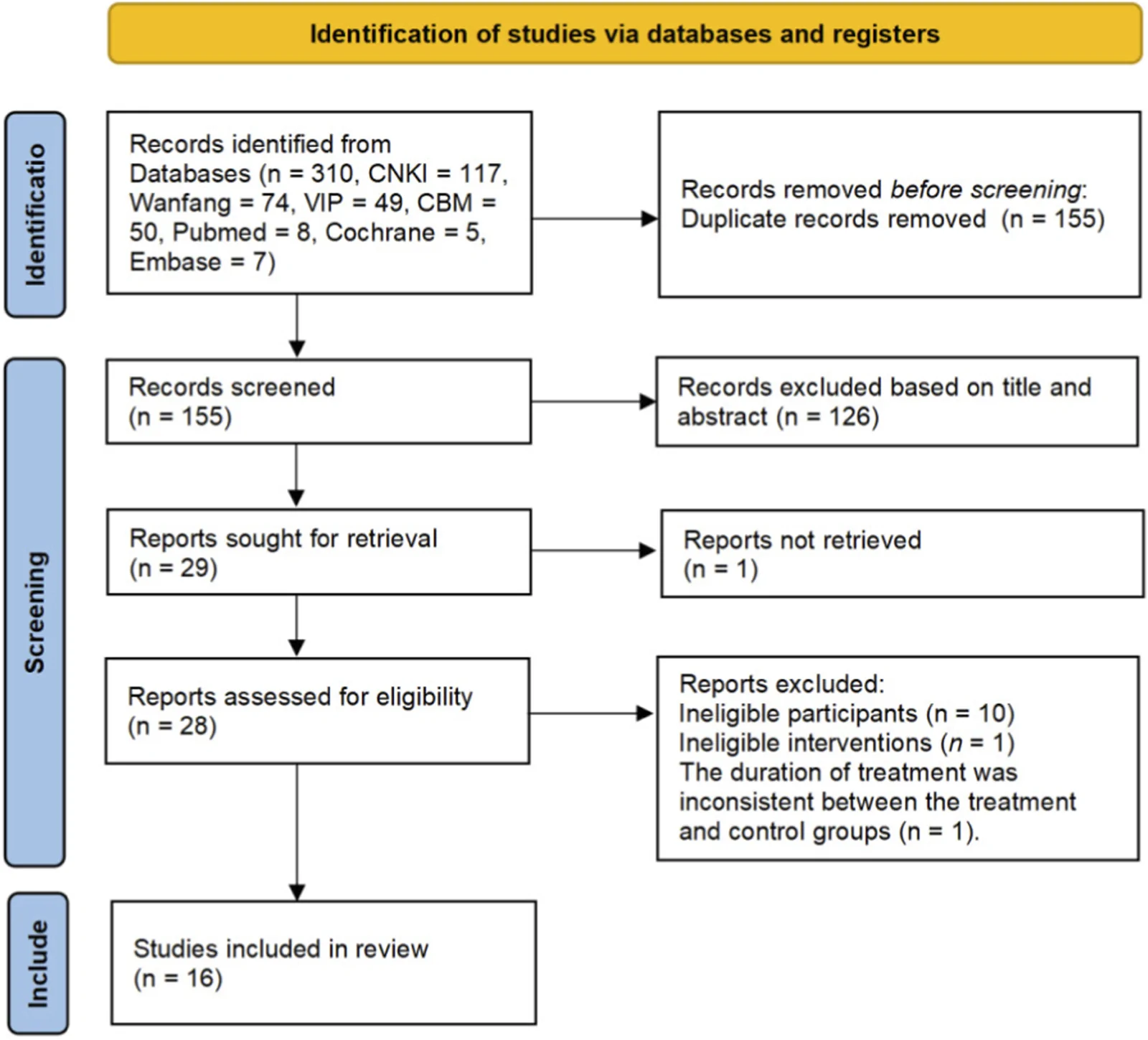
PRISMA flow diagram of literature screening process.

### 3.2 Study characteristics

A total of 16 RCTs involving 1,391 participants were included in this review, with 697 patients in the experimental group and 694 in the control group. The sample sizes ranged from 40 to 148 per study. All studies were parallel-design RCTs conducted in hospital settings in China. All studies compared QLQX plus WM with the same WM alone. The control groups received various WM regimens, including anticoagulants, antiplatelet agents, β-blockers, vasodilators, lipid-lowering agents, and other cardiovascular medications. The specific background treatments reported in each trial and their between-group comparability are detailed in Supplementary Material 4. Treatment duration across studies ranged from 3 weeks to 6 months. Detailed characteristics of the included studies are presented in Table 1.

**TABLE 1:** Characteristics of included studies.

Study	Sample size		Age (years)		Treatment		Treatment duration	Outcome
	I	C	I	C	I	C		
Bai LQ 2013 (Bai et al., 2013)	20	20	45 ± 3.7	52 ± 4.2	QLQX 1.2 g tid + WM	WM	3 m	③⑧
Chen SH 2019 (Chen, 2019)	30	30	67.1 ± 6.9	56.8 ± 6.7	QLQX 1.2 g tid + WM	WM	1 m	⑧
Chen YQ 2020 (Chen et al., 2020a)	51	51	63.16 ± 11.57	62.85 ± 11.73	QLQX 1.2 g tid + WM	WM	6 m	①③⑤⑧
Chen YZ 2025 (Chen, 2025)	50	50	62.48 ± 8.67	62.13 ± 8.25	QLQX 1.2 g tid + WM	WM	6 m	①③⑥⑧⑨⑪
Han L2024 (Han et al., 2024)	46	46	68.45 ± 3.42	68.34 ± 3.31	QLQX 1.2 g tid + WM	WM	3 m	①③④⑤⑦⑧
Hou XL 2010 (Hou, 2010)	46	45	55 ± 4.7	52 ± 3.2	QLQX 1.2 g tid + WM	WM	3 m	③⑥⑧
Liu YJ 2012 (Liu, 2012)	30	30	78–90	76–92	QLQX 1.2 g tid + WM	WM	30 days (1 m)	③⑧
Liu Y2023 (Liu et al., 2023)	41	41	71.58 ± 8.46	70.34 ± 9.15	QLQX 1.2 g tid + WM	WM	3w	①③⑦⑧⑩
Luo F2019 (Luo, 2019)	31	29	56.97 ± 8.82	57.14 ± 8.66	QLQX 1.2 g tid + WM	WM	3 m	③④⑤⑧
Pang J2014 (Pang et al., 2014)	55	55	62.8 ± 7.7	63.2 ± 7.5	QLQX 1.2 g tid + WM	WM	3 m	②③⑤⑦⑧
Qi MJ 2024 (Qi et al., 2024)	37	37	60.46 ± 8.45	61.65 ± 9.99	QLQX 1.2 g tid + WM	WM	3 m	⑦⑧
Qiao DF 2018 (Qiao, 2018)	61	61	54.5 ± 2.2	54.3 ± 2.7	QLQX 1.2 g tid + WM	WM	3 m	③④⑤⑧
Shi XL 2024 (Shi, 2024)	45	45	62.67 ± 6.55	62.53 ± 6.41	QLQX 1.2 g tid + WM	WM	4w (1 m)	③④⑪
Wang ZJ 2015 (Wang et al., 2015)	74	74	NR	NR	QLQX 1.2 g tid + WM	WM	3 m	①③⑦⑧
Xi K2011 (Xi et al., 2011)	30	30	NR	NR	QLQX 1.2 g tid + WM	WM	4w (1 m)	⑧
Xie ZQ 2022 (Xie et al., 2022)	50	50	71.14 ± 2.08	71.25 ± 2.02	QLQX 1.2 g tid + WM	WM	3 m	①③⑧⑪

① N-terminal pro-B-type natriuretic peptide (NT-proBNP) ② B-type natriuretic peptide (BNP) ③ left ventricular ejection fraction (LVEF) ④ left ventricular end-systolic diameter (LVESD) ⑤ left ventricular end-diastolic diameter (LVEDD) ⑥ left ventricular end-diastolic volume (LVEDV) ⑦ high-sensitivity C-reactive protein (hs-CRP) ⑧ clinical response rate ⑨ quality of life ⑩ all cause mortality ⑪ adverse events.

C, control group; d, day; I, intervention group; m, month; NR, not reported; QLQX, qili qiangxin capsule; tid, three times daily; WM, western medicine; w, week.

### 3.3 Risk of bias in included studies

Among the included 16 RCTs, nine studies reported using a random number table to generate the allocation sequence (Chen, 2019; Luo, 2019; Chen et al., 2020a; Xie et al., 2022; Liu et al., 2023; Han et al., 2024; Qi et al., 2024; Shi, 2024; Chen, 2025), and the remaining 7 studies did not report the method used for sequence generation. None of the studies reported allocation concealment. None of the studies reported blinding of participants, personnel, or outcome assessors. Only one study reported dropouts (Pang et al., 2014), but the missing outcome data were minimal. No dropouts were reported in the remaining studies. No study reported registration information. According to the RoB 2 assessment, all 16 studies had some concerns regarding the randomization process, deviations from intended interventions, and selection of the reported result, whereas the risk of bias due to missing outcome data was judged to be low in all studies. For measurement of the outcome, 14 studies had some concerns and 2 studies were judged to have a high risk of bias. These two studies reported only the clinical response rate, which is a relatively subjective outcome, contributing to a high risk of bias in the measurement of the outcome domain and resulting in an overall high risk of bias. Overall, 14 studies were judged to have some concerns and 2 studies were judged to have a high risk of bias. The results of the risk of bias assessment are shown in Figures 2, 3.

**FIGURE 2:**
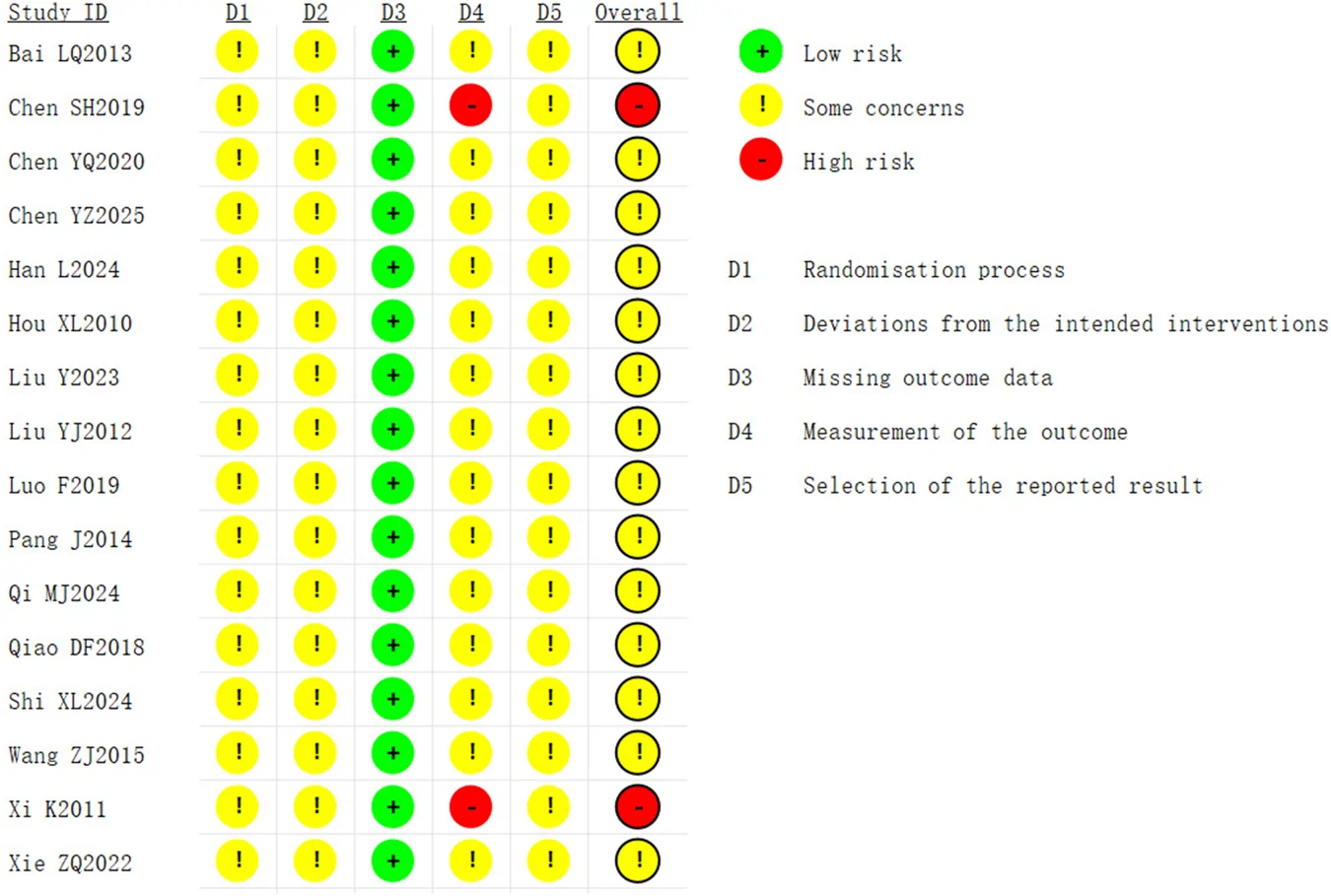
Risk of bias summary.

**FIGURE 3:**
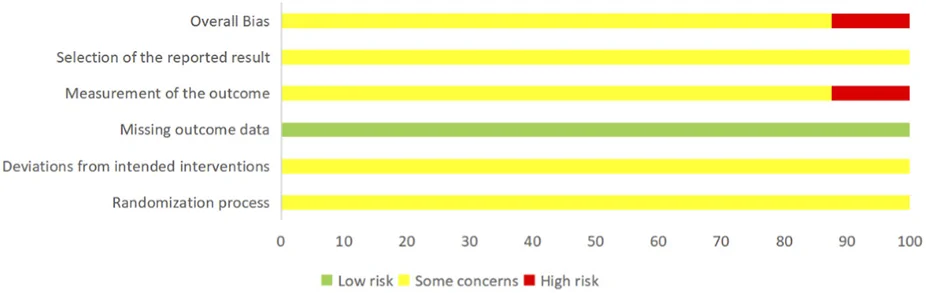
Risk of bias graph.

### 3.4 Effect estimates

#### 3.4.1 NT-proBNP

Six RCTs reported NT-proBNP (Wang et al., 2015; Chen et al., 2020a; Xie et al., 2022; Liu et al., 2023; Han et al., 2024; Chen, 2025), including a total of 624 participants, with 312 in the experimental group and 312 in the control group. Because NT-proBNP was reported using different numerical scales across the included studies, the SMD was used to pool the results. The meta-analysis showed that QLQX combined with WM was associated with lower NT-proBNP levels than WM alone (SMD = −1.14, 95% CI [-1.74, −0.54], *P* = 0.0002), as shown in Figure 4. Substantial heterogeneity was observed among the included studies. Given the limited and inconsistently reported study-level information, subgroup analysis was conducted according to treatment duration as the only prespecified factor that could be meaningfully examined. However, treatment duration did not explain the observed heterogeneity. Other potential sources, including baseline HF severity, differences in WM regimens and dosages, and other clinical characteristics, could not be reliably assessed because of insufficient reporting. Therefore, the pooled effect estimate should be interpreted with caution. The detailed results of subgroup analysis are presented in Supplementary Material 5.

**FIGURE 4:**
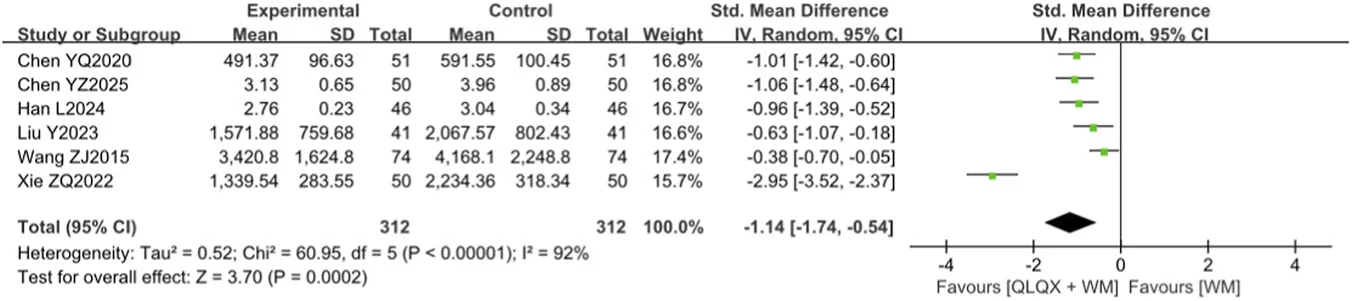
Forest plot of NT-proBNP for QLQX + WM vs. WM.

#### 3.4.2 BNP

One RCT (Pang et al., 2014) (N = 107, 53 cases in the experimental group, 54 cases in the control group) showed that QLQX combined with WM was more effective in reducing BNP compared to WM alone (MD = −144.10 ng/L, 95% CI [-196.97, −91.23], *P* < 0.00001).

#### 3.4.3 LVEF

A total of 13 RCTs reported LVEF (Hou, 2010; Liu, 2012; Bai et al., 2013; Pang et al., 2014; Wang et al., 2015; Qiao, 2018; Luo, 2019; Chen et al., 2020a; Xie et al., 2022; Liu et al., 2023; Han et al., 2024; Shi, 2024; Chen, 2025), including 1,194 participants, with 598 in the experimental group and 596 in the control group. The meta-analysis showed that QLQX combined with WM was more effective in improving LVEF compared to WM alone (MD = 6.04%, 95% CI [4.77, 7.31], *P* < 0.00001), as shown in Figure 5. The heterogeneity test indicated the presence of heterogeneity. We conducted subgroup analysis based on the treatment duration, and the results showed that the factor was not the source of heterogeneity. The detailed results of subgroup analysis are presented in Supplement 5.

**FIGURE 5:**
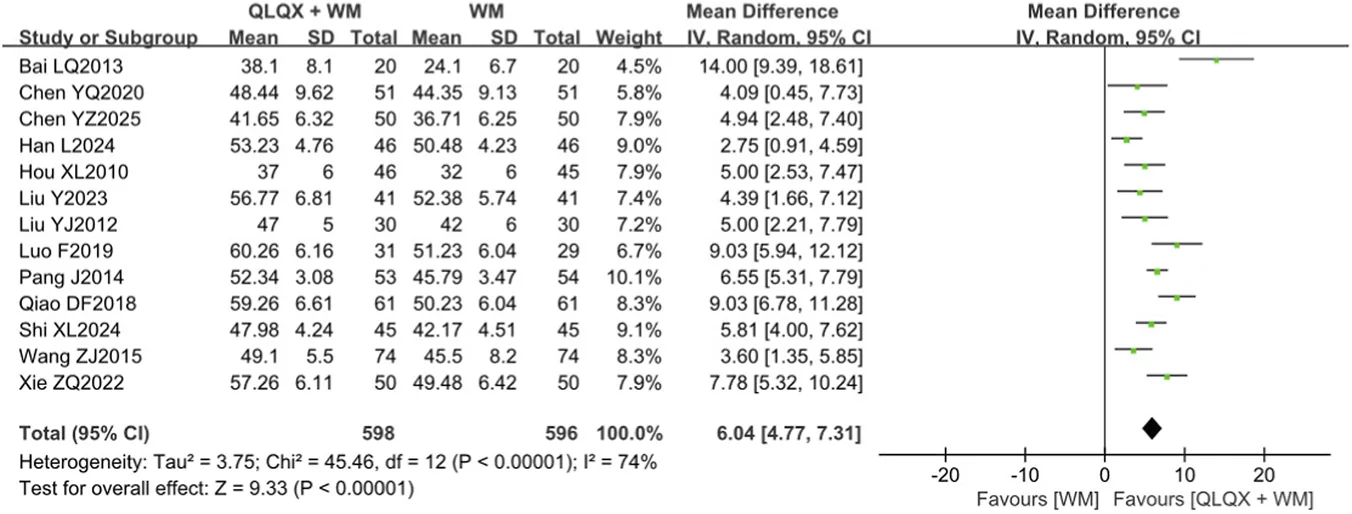
Forest plot of LVEF for QLQX + WM vs. WM.

#### 3.4.4 LVESD

Four RCTs reported LVESD (Qiao, 2018; Luo, 2019; Han et al., 2024; Shi, 2024), including a total of 364 participants, with 183 in the experimental group and 181 in the control group. The meta-analysis showed that QLQX combined with WM was more effective than WM alone in reducing LVESD (MD = −5.80 mm, 95% CI [-8.77, −2.82], *P* = 0.0001), as shown in Figure 6. The heterogeneity test indicated the presence of heterogeneity. We performed a subgroup analysis by treatment duration but did not identify the source of heterogeneity. The detailed results of subgroup analysis are presented in Supplement 5.

**FIGURE 6:**
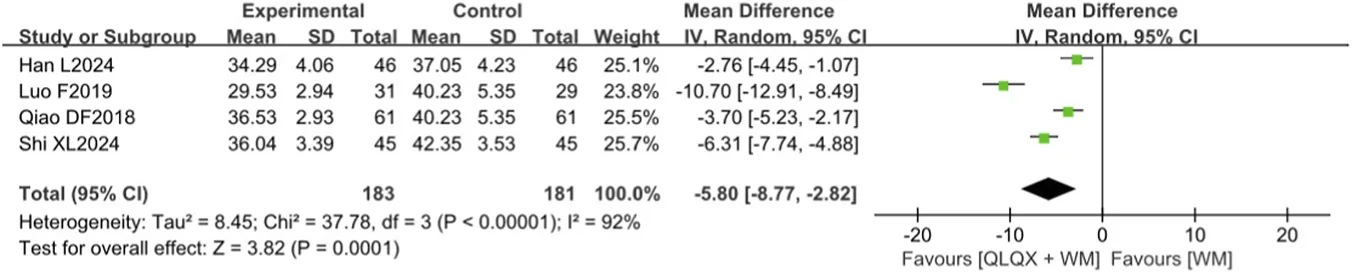
Forest plot of LVESD for QLQX + WM vs. WM.

#### 3.4.5 LVEDD

Five RCTs reported LVEDD (Pang et al., 2014; Qiao, 2018; Luo, 2019; Chen et al., 2020a; Han et al., 2024), including a total of 483 participants, with 242 in the experimental group and 241 in the control group. The meta-analysis showed that QLQX combined with WM was more effective than WM alone in reducing LVEDD (MD = −6.86 mm, 95% CI [-9.52, −4.21], *P* < 0.00001), as shown in Figure 7. Heterogeneity was detected by the heterogeneity test. However, subgroup analysis based on treatment duration did not reveal the source of this heterogeneity. The detailed results of subgroup analysis are presented in Supplement 5.

**FIGURE 7:**
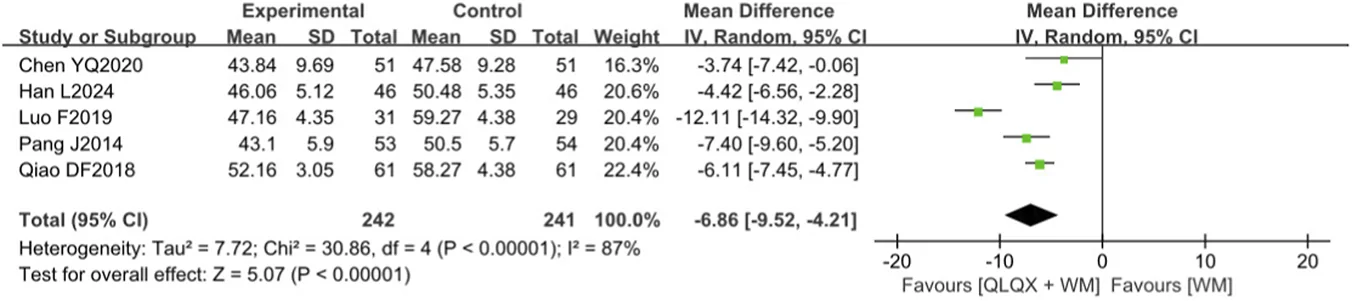
Forest plot of LVEDD for QLQX + WM vs. WM.

#### 3.4.6 LVEDV

Two RCTs reported LVEDV (Hou, 2010; Chen, 2025), including a total of 191 participants, with 96 in the experimental group and 95 in the control group. The meta-analysis showed no statistically difference in effectiveness between QLQX combined with WM and WM alone in reducing LVEDV (MD = −13.98 mL, 95% CI [-30.12, 2.16], *P* = 0.09), as shown in Figure 8.

**FIGURE 8:**
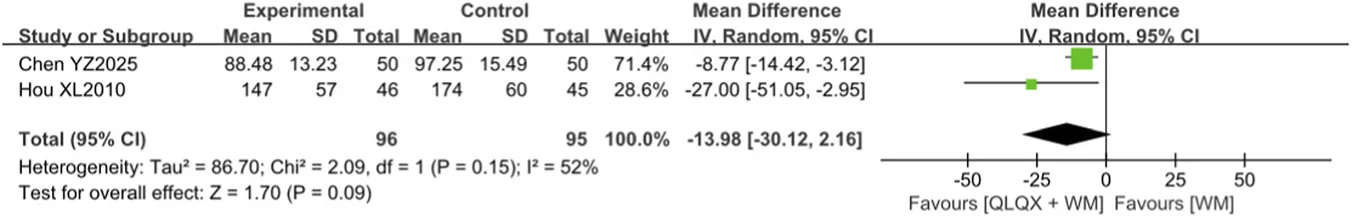
Forest plot of LVEDV for QLQX + WM vs. WM.

#### 3.4.7 hs-CRP

Five RCTs reported hs-CRP (Pang et al., 2014; Wang et al., 2015; Liu et al., 2023; Han et al., 2024; Qi et al., 2024), including a total of 503 participants, with 251 in the experimental group and 252 in the control group. The meta-analysis showed that QLQX combined with WM was more effective than WM alone in reducing hs-CRP (MD = −3.50 mg/L, 95% CI [-5.74, −1.27], *P* = 0.002), as shown in Figure 9. Substantial heterogeneity was observed. We conducted subgroup analysis considering the treatment duration, and the results showed that the factor was not the source of heterogeneity. The detailed results of subgroup analysis are presented in Supplement 5.

**FIGURE 9:**
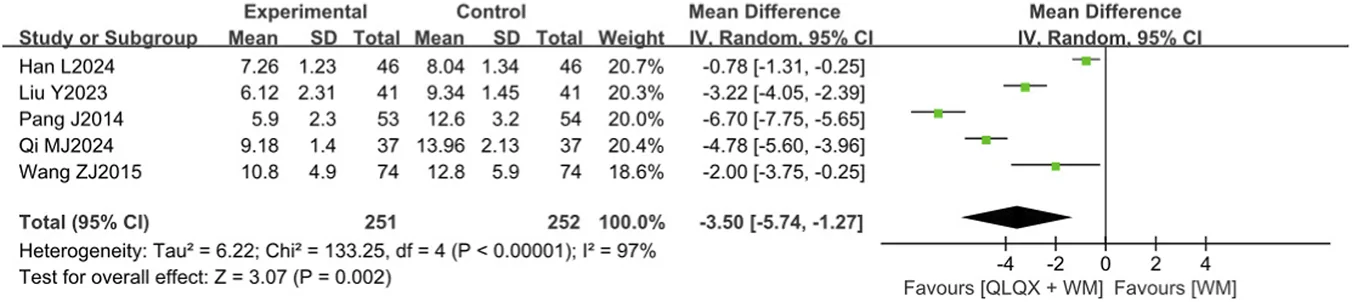
Forest plot of hs-CRP for QLQX + WM vs. WM.

#### 3.4.8 Clinical response rate

A total of 15 RCTs reported clinical response rate (Hou, 2010; Xi et al., 2011; Liu, 2012; Bai et al., 2013; Pang et al., 2014; Wang et al., 2015; Qiao, 2018; Chen, 2019; Luo, 2019; Chen et al., 2020a; Xie et al., 2022; Liu et al., 2023; Han et al., 2024; Qi et al., 2024; Chen, 2025), including 1,298 participants, with 650 in the experimental group and 648 in the control group. The criteria used to define clinical response varied across studies. Most studies primarily based treatment response on improvements in cardiac function classification, while some additionally considered changes in clinical symptoms, physical signs, LVEF, or TCM syndrome scores, or used the Killip classification as the primary basis for assessment. The meta-analysis showed that QLQX combined with WM was more effective than WM alone in improving clinical response rate (RR = 1.21, 95% CI [1.16, 1.27], *P* < 0.00001), as shown in Figure 10.

**FIGURE 10:**
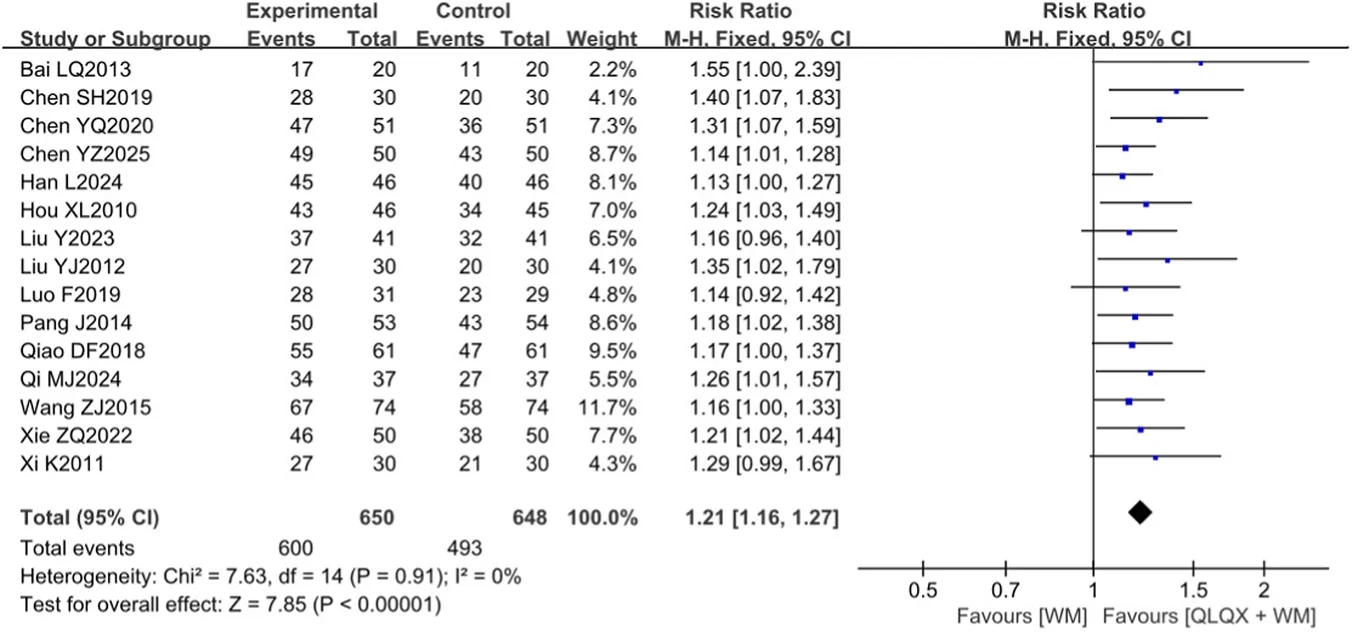
Forest plot of clinical response rate for QLQX + WM vs. WM.

#### 3.4.9 Quality of life

One RCT (Chen, 2025) (N = 100, 50 cases in the experimental group, 50 cases in the control group) showed that QLQX combined with WM was more effective in improving MLHFQ compared to WM alone (MD = −7.64, 95% CI [-10.32, −4.96], *P* < 0.00001).

#### 3.4.10 All-cause mortality

One RCT (Liu et al., 2023) (N = 82, 41 cases in the experimental group, 41 cases in the control group) showed no statistically difference in all-cause mortality between QLQX plus WM and WM alone (RR = 0.30, 95% CI [0.09, 1.01], *P* = 0.05).

#### 3.4.11 Adverse events

Three RCTs reported adverse events (Xie et al., 2022; Shi, 2024; Chen, 2025), including a total of 290 participants, with 145 in the experimental group and 145 in the control group. The meta-analysis showed no statistically difference in adverse events between QLQX plus WM and WM alone (RR = 1.08, 95% CI [0.51, 2.28], *P* = 0.83), as shown in Figure 11.

**FIGURE 11:**
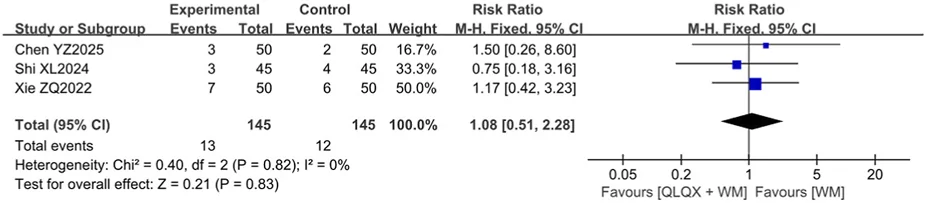
Forest plot of adverse events for QLQX + WM vs. WM.

#### 3.4.12 Heart failure‒related hospitalisation

No study reported the outcome of heart failure‒related hospitalisation.

### 3.5 Sensitivity analysis

Sensitivity analyses were conducted using two approaches. First, for the primary outcome, NT-proBNP, sequential exclusion of individual studies did not materially alter the pooled effect estimate, and the overall conclusion remained unchanged, suggesting that the result was robust to the influence of individual studies, as shown in Figure 12. Second, a risk-of-bias-based sensitivity analysis was performed for the clinical response rate by excluding the two studies judged to have an overall high risk of bias (Chen SH2019 and Xi K2011). The pooled effect remained statistically significant after exclusion (RR = 1.20, 95% CI [1.14, 1.26], *P* < 0.00001), with no observed heterogeneity (*I* ^2^ = 0%). These findings were consistent with the primary analysis (RR = 1.21, 95% CI [1.16, 1.27], *P* < 0.00001), indicating that the overall effect estimate was not materially influenced by the two studies at high risk of bias.

**FIGURE 12:**
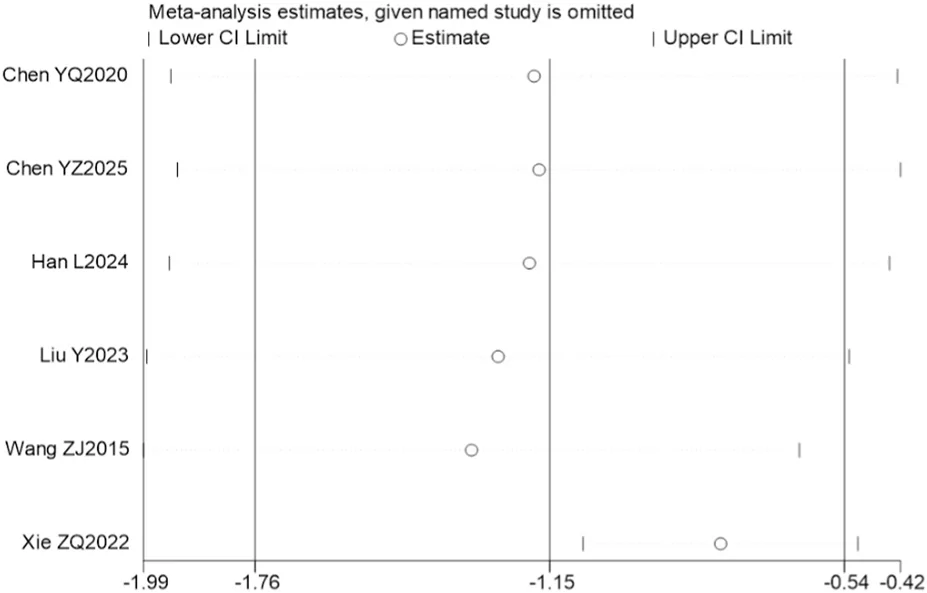
Sensitivity analysis results for NT-proBNP.

### 3.6 Publication bias

Publication bias was assessed using funnel plots when the number of RCTs for a single outcome measure was greater than 10. The results showed asymmetry in the funnel plots, indicating the potential presence of publication bias. See Figure 13.

**FIGURE 13:**
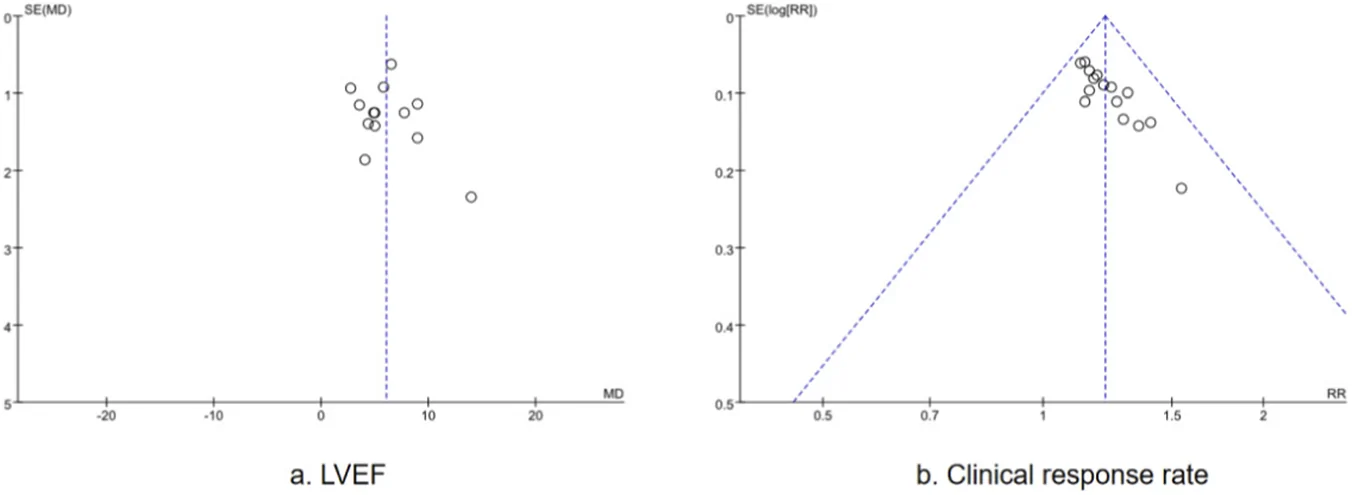
Funnel plots for **(a)** LVEF and **(b)** clinical response rate.

### 3.7 GRADE certainty of evidence

According to the GRADE approach, the certainty of evidence for all outcomes ranged from low to very low. The certainty of evidence for each outcome was downgraded mainly due to concerns about risk of bias, inconsistency, imprecision, and publication bias. Table 2 presents details of the evaluation of the evidence certainty for outcomes.

**TABLE 2:** Summary of evidence certainty for outcomes.

Outcome	No. of studies	Risk of bias	Inconsistency	Indirectness	Imprecision	Other considerations	No. of patients		Effect	Certainty
							I	C		
NT-proBNP	6	Serious^1^	Serious^2^	Not serious	Not serious	None	312	312	SMD -1.14 (−1.74 to −0.54)	Low (⊕⊕○○)
BNP (ng/L)	1	Serious^1^	NA	Not serious	Serious^3^	None	53	54	MD -144.10 (−196.97 to −91.23)	Low (⊕⊕○○)
LVEF (%)	13	Serious^1^	Serious^2^	Not serious	Not serious	None	598	596	MD 6.04 (4.77–7.31)	Low (⊕⊕○○)
LVESD (mm)	4	Serious^1^	Serious^2^	Not serious	Serious^3^	None	183	181	MD -5.80 (−8.77 to −2.82)	Very low (⊕○○○)
LVEDD (mm)	5	Serious^1^	Serious^2^	Not serious	Not serious	None	242	241	MD -6.86 (−9.52 to −4.21)	Low (⊕⊕○○)
LVEDV (ml)	2	Serious^1^	Serious^2^	Not serious	Serious^3^	None	96	95	MD -13.98 (−30.12 to 2.16)	Very low (⊕○○○)
hs-CRP (mg/L)	5	Serious^1^	Serious^2^	Not serious	Not serious	None	251	252	MD -3.50 (−5.74 to −1.27)	Low (⊕⊕○○)
Clinical response rate	15	Serious^1^	Not serious	Not serious	Not serious	publication bias strongly suspected^4^	600/650	493/648	RR 1.21 (1.16–1.27)	Low (⊕⊕○○)
Quality of life (MLHFQ)	1	Serious^1^	NA	Not serious	Serious^3^	None	50	50	MD -7.64 (−10.32 to −4.96)	Low (⊕⊕○○)
All-cause mortality	1	Serious^1^	NA	Not serious	Serious^3^	None	41	41	RR 0.30 (0.09–1.01)	Low (⊕⊕○○)
Adverse events	3	Serious^1^	Not serious	Not serious	Serious^5^	None	13/145	12/145	RR 1.08 (0.51–2.28)	Low (⊕⊕○○)

^1^Lack of concealment of the allocation; blinding method was not implemented,^2^ *I* ^2^ ≥ 50%, ^3^The total population size is less than 400, ^4^A funnel plot indicates potential publication bias, ^5^The total number of events is less than 300.

C, control group; I, intervention group; MD, mean difference; NA, not applicable; RR, relative risk.

## 4 Discussion

### 4.1 Summary of main results in this review

This study compared patients with HF after AMI receiving WM with and without QLQX, synthesising the current evidence on the clinical use of QLQX in this population. A total of 16 RCTs, involving 1391 participants from China, were included. The findings suggest that QLQX combined with WM was associated with lower NT-proBNP and BNP levels and higher LVEF than WM alone. In addition, QLQX was associated with reductions in LVESD, LVEDD, and hs-CRP, suggesting potential benefits in cardiac function, ventricular remodelling, and inflammatory status. The pooled results also demonstrated a higher clinical response rate and improved quality of life in patients receiving QLQX. However, no statistically difference was observed for LVEDV. Regarding safety, the incidence of adverse events did not differ significantly between groups, indicating that the addition of QLQX to WM was generally well tolerated. However, evidence for hard clinical endpoints was extremely limited. Only one small RCT reported all-cause mortality, with no statistically significant difference between groups, while no study reported heart failure-related hospitalisation. Therefore, whether the observed improvements in surrogate outcomes translate into reductions in mortality or rehospitalisation remains uncertain.

### 4.2 Potential mechanisms of QLQX for HF after AMI

The potential benefits of QLQX observed in the present study are broadly consistent with the clinical evidence in the wider HF population. A recent comprehensive review summarized evidence that QLQX may improve cardiac function and clinical outcomes in patients with chronic HF, with its effects potentially related to the modulation of cardiac remodeling, inflammation, fibrosis, neurohormonal activation, and myocardial metabolism (Cheang et al., 2025). Similarly, the QUEST trial demonstrated that QLQX reduced the risk of heart failure hospitalization or cardiovascular death in patients with HFrEF, with generally consistent effects in patients with an ischemic etiology (Cheang et al., 2024). These findings, together with our results, suggest that the potential benefits of QLQX may extend to patients with different HF backgrounds. However, the present study provides more specific evidence for HF developing after AMI.

The beneficial effects of QLQX in HF after AMI may involve multiple pathological processes, particularly ventricular remodeling, cardiomyocyte apoptosis, and inflammation. Ventricular remodeling is a major determinant of HF progression after AMI. In the present review, QLQX improved LVEF and reduced LVEDD and LVESD, suggesting a protective effect against adverse cardiac remodeling. Experimental studies have shown that QLQX attenuates myocardial fibrosis, regulates collagen homeostasis, and promotes angiogenesis after AMI by suppressing remodeling-related factors such as TGF-β1, MMP-9, and α-SMA, thereby contributing to improvements in cardiac structure and function (Sun et al., 2024). Cardiomyocyte apoptosis and inflammation are also critical drivers of HF after AMI. Previous studies have demonstrated that QLQX protects cardiomyocytes by reducing apoptosis and excessive autophagy through pathways including PI3K/Akt and ROS/AMPK/mTOR signaling (Fan et al., 2022; Wu et al., 2025). In addition, QLQX has been shown to suppress inflammatory responses by inhibiting the TMAO/NF-κB pathway and reducing the expression of pro-inflammatory cytokines such as TNF-α, IL-6, and IL-1β (Jiao et al., 2025). These mechanisms are consistent with the reductions in NT-proBNP, BNP, and hs-CRP observed in the present review. Overall, the available evidence suggests that QLQX may exert coordinated anti-remodeling, anti-apoptotic, and anti-inflammatory effects, thereby contributing to improved cardiac function and clinical outcomes in patients with HF after AMI.

### 4.3 Implications for future research

Based on the current research findings, QLQX may provide additional benefits for patients with HF after AMI. However, the existing evidence still carries uncertainty. Therefore, future research should focus on generating higher-quality evidence. First, large-scale, multicentre RCTs with rigorous methodology are needed. Future studies should clearly describe randomisation procedures, allocation concealment, and blinding to reduce the risk of bias and improve the reliability of the findings. Second, studies should adopt longer follow-up periods and report clinically meaningful endpoints, such as all-cause mortality and heart failure-related hospitalisation. Third, greater standardization of conventional Western medical therapy and QLQX treatment protocols is warranted. Variations in background therapy, treatment duration, and outcome definitions across studies may have contributed to the observed heterogeneity. Overall, future research should aim to generate high-certainty evidence through well-designed trials with robust methodology, standardized outcomes, longer follow-up, and clinically meaningful endpoints, while also further elucidating the mechanisms underlying the potential benefits of QLQX in HF after AMI.

### 4.4 Strengths and limitations

This review has several strengths. To the best of our knowledge, this is the first GRADE-assessed systematic review and meta-analysis specifically evaluating the efficacy and safety of QLQX as an adjunctive therapy in patients with HF after AMI. We identified one previous systematic review and meta-analysis evaluating QLQX in patients with AMI complicated by HF (Chen et al., 2020b). However, the study population was not restricted to patients who developed HF secondary to AMI, which differs from the clinical condition examined in the present review. By focusing specifically on HF after AMI, our study provides more targeted evidence for this high-risk patient population. An important consideration is the recently published QUEST trial, a large, multicentre, double-blind, placebo-controlled RCT evaluating QLQX in patients with HFrEF (Cheang et al., 2024). The trial reported a significant reduction in the composite of heart failure hospitalisation or cardiovascular death, with treatment effects generally consistent in the prespecified subgroup with an ischaemic aetiology. However, we did not include the ischaemic subgroup from QUEST in the present meta-analysis because an ischaemic aetiology does not necessarily indicate that HF developed secondary to a prior AMI. Including this subgroup would therefore have introduced indirectness and altered the predefined study population. Nevertheless, the findings from QUEST provide important complementary evidence regarding the potential benefit of QLQX in patients with HFrEF of ischaemic aetiology and are directionally consistent with some of our findings. In addition, we conducted a comprehensive search across seven databases to ensure the inclusion of all relevant RCTs. A language-unrestricted search strategy was employed to minimize selection bias, and the methodology was reported in detail and transparently to allow for replication. Additionally, the certainty of evidence was assessed using the GRADE approach, enabling a more transparent evaluation of the strength and reliability of the findings (Robinson and Liu, 2025; Zhang et al., 2025).

Several limitations should also be acknowledged. First, the methodological quality of the included studies was generally limited. None of the studies reported allocation concealment or blinding, and seven studies did not adequately describe the randomization process. These methodological limitations raised concerns about bias, particularly in the randomization and measurement of outcomes, and may have led to overestimation of the treatment effects. Therefore, the findings from these trials should be interpreted in light of the potential risk of bias. Second, several outcomes were based on small sample sizes, limiting the precision and robustness of the pooled estimates. Third, substantial heterogeneity was observed for several outcomes, particularly the primary outcome of NT-proBNP. Although subgroup analysis based on treatment duration was conducted, this factor did not explain the heterogeneity. Other potentially important sources, such as baseline HF severity, differences in concomitant WM regimens and dosages, and other clinical characteristics, could not be adequately explored because of insufficient and inconsistent reporting across the included studies. Importantly, the use of a random-effects model accounts for between-study variation but does not eliminate the underlying clinical or methodological heterogeneity. Therefore, the pooled estimates, particularly for NT-proBNP, should be interpreted cautiously. In addition, the pooled NT-proBNP effect was expressed as a SMD because the reported numerical scales and measurement units differed across the included studies. The SMD represents a standardised between-group difference rather than an absolute change in NT-proBNP concentration. Therefore, it should not be interpreted as a specific reduction in NT-proBNP in clinical units. Fourth, there was a deviation from the prespecified outcome data format. The registered protocol specified change in NT-proBNP from baseline to the end of treatment as the primary outcome. However, none of the included studies report change-from-baseline values, and the available information was insufficient to reliably derive the standard deviation of change without making assumptions regarding the correlation between baseline and post-treatment measurements. Therefore, end-of-treatment values were analysed instead. Although this approach allowed the available data to be synthesised without relying on imputed correlations, the difference between the prespecified and actual data format should be acknowledged. Fifth, the evidence for clinically important hard endpoints was particularly limited. Only one small RCT reported all-cause mortality, and none reported heart failure-related hospitalisation. Most statistically significant outcomes in this review, including NT-proBNP, LVEF, LVEDD, LVESD, and hs-CRP, were surrogate or intermediate outcomes. Although these measures are clinically relevant for assessing cardiac function and ventricular remodeling, improvements in surrogate outcomes cannot be assumed to translate into reductions in mortality or rehospitalisation. Therefore, the present findings provide insufficient evidence to determine whether QLQX improves long-term clinical prognosis. In addition, the clinical response rate was defined differently across studies and involved relatively subjective assessments, which may limit the interpretability of this outcome. Most studies assessed treatment response primarily according to changes in cardiac function, particularly improvements in NYHA or other heart failure functional classifications, several studies additionally incorporated changes in symptoms and physical signs, while others included more specific measures such as LVEF or TCM syndrome scores, and some studies used Killip classification or other investigator-defined criteria. Therefore, although the pooled analysis showed a higher clinical response rate with QLQX plus WM, the subjective nature and heterogeneous definitions of this outcome may have introduced measurement bias and reduced the comparability of the estimates. This finding should therefore be interpreted cautiously. Moreover, all included studies were conducted in China, which may limit the generalisability of the findings to other populations and healthcare settings. Overall, these limitations substantially reduce confidence in the current evidence, and the findings should therefore be regarded as hypothesis-generating rather than definitive evidence of efficacy.

## 5 Conclusion

This study found that QLQX plus conventional WM was associated with improvements in several surrogate and short-term clinical outcomes in patients with HF after AMI. However, the evidence was of low or very low certainty, and data on clinically important outcomes such as mortality and heart failure-related hospitalisation were insufficient. Therefore, the current evidence does not establish whether QLQX improves long-term clinical prognosis, and it should not be considered sufficient to inform changes in clinical practice or guideline recommendations. Further well-designed, adequately powered RCTs with longer follow-up and clinically important endpoints are warranted.

## Data Availability

The original contributions presented in the study are included in the article/Supplementary Material, further inquiries can be directed to the corresponding author.
