# Supplementary material for: Qili Qiangxin capsule as adjunctive therapy for heart failure after acute myocardial infarction: a GRADE-assessed systematic review and meta-analysis of randomised controlled trials
Source: Front Pharmacol. 2026 Oct 19;17:1959187. doi: 10.3389/fphar.2026.1959187 (PMC13724362; doi:10.3389/fphar.2026.1959187)
Supplement: Supplementary file 1 [file DataSheet1.docx]

**Supplement 1. PRISMA 2020 Checklist.**

| Section and Topic | Item # | Checklist item | **Location where item is reported** |
| --- | --- | --- | --- |
| TITLE | | |  |
| Title | 1 | Identify the report as a systematic review. | **Title** |
| ABSTRACT | | |  |
| Abstract  Structured summary | 2 | See the PRISMA 2020 for Abstracts checklist.  Background: main objectives  Methods: data sources; study eligibility criteria, participants, and interventions; study appraisal; and synthesis methods, such as network meta-analysis.  Results: number of studies and participants identified; summary estimates with corresponding confidence/credible intervals; treatment rankings may also be discussed. Authors may choose to summarize pairwise comparisons against a chosen treatment included in their analyses for brevity.  Discussion/Conclusions: limitations; conclusions and implications of findings.  Other: primary source of funding; systematic review registration number with registry name. | **Abstract** |
| INTRODUCTION | | |  |
| Rationale | 3 | Describe the rationale for the review in the context of existing knowledge. | **1. Introduction** |
| Objectives | 4 | Provide an explicit statement of the objective(s) or question(s) the review addresses. | **1. Introduction** |
| METHODS | | |  |
| Eligibility criteria | 5 | Specify the inclusion and exclusion criteria for the review and how studies were grouped for the syntheses. | **2. Methods; 2.2. Inclusion criteria; 2.3. Exclusion criteria** |
| Information sources | 6 | Specify all databases, registers, websites, organisations, reference lists and other sources searched or consulted to identify studies. Specify the date when each source was last searched or consulted. | **2. Methods; 2.1. Search strategy** |
| Search strategy | 7 | Present the full search strategies for all databases, registers and websites, including any filters and limits used. | **2. Methods; 2.1. Search strategy** |
| Selection process | 8 | Specify the methods used to decide whether a study met the inclusion criteria of the review, including how many reviewers screened each record and each report retrieved, whether they worked independently, and if applicable, details of automation tools used in the process. | **2. Methods;** **2.4. Study selection and data extraction** |
| Data collection process | 9 | Specify the methods used to collect data from reports, including how many reviewers collected data from each report, whether they worked independently, any processes for obtaining or confirming data from study investigators, and if applicable, details of automation tools used in the process. | **2. Methods; 2.4. Study selection and data extraction** |
| Data items | 10a | List and define all outcomes for which data were sought. Specify whether all results that were compatible with each outcome domain in each study were sought (e.g. for all measures, time points, analyses), and if not, the methods used to decide which results to collect. | **2. Methods; 2.2. Inclusion criteria; 2.3. Exclusion criteria; 2.4. Study selection and data extraction** |
|  | 10b | List and define all other variables for which data were sought (e.g. participant and intervention characteristics, funding sources). Describe any assumptions made about any missing or unclear information. | **2. Methods; 2.4. Study selection and data extraction** |
| Study risk of bias assessment | 11 | Specify the methods used to assess risk of bias in the included studies, including details of the tool(s) used, how many reviewers assessed each study and whether they worked independently, and if applicable, details of automation tools used in the process. | **2. Methods;** **2.5. Trial quality assessment** |
| Effect measures | 12 | Specify for each outcome the effect measure(s) (e.g. risk ratio, mean difference) used in the synthesis or presentation of results. | **2. Methods;** **2.6. Data synthesis** |
| Synthesis methods | 13a | Describe the processes used to decide which studies were eligible for each synthesis (e.g. tabulating the study intervention characteristics and comparing against the planned groups for each synthesis (item #5)). | **2. Methods; 2.6. Data synthesis** |
|  | 13b | Describe any methods required to prepare the data for presentation or synthesis, such as handling of missing summary statistics, or data conversions. | **2. Methods; 2.6. Data synthesis** |
|  | 13c | Describe any methods used to tabulate or visually display results of individual studies and syntheses. | **2. Methods; 2.6. Data synthesis** |
|  | 13d | Describe any methods used to synthesize results and provide a rationale for the choice(s). If meta-analysis was performed, describe the model(s), method(s) to identify the presence and extent of statistical heterogeneity, and software package(s) used. | **2. Methods; 2.6. Data synthesis** |
|  | 13e | Describe any methods used to explore possible causes of heterogeneity among study results (e.g. subgroup analysis, meta-regression). | **2. Methods; 2.6. Data synthesis** |
|  | 13f | Describe any sensitivity analyses conducted to assess robustness of the synthesized results. | **2. Methods; 2.6. Data synthesis** |
| Reporting bias assessment | 14 | Describe any methods used to assess risk of bias due to missing results in a synthesis (arising from reporting biases). | **2. Methods; 2.5. Data synthesis** |
| Certainty assessment | 15 | Describe any methods used to assess certainty (or confidence) in the body of evidence for an outcome. | **2. Methods; 2.7. GRADE certainty assessment of the evidence** |
| RESULTS | | |  |
| Study selection | 16a | Describe the results of the search and selection process, from the number of records identified in the search to the number of studies included in the review, ideally using a flow diagram. | **3. Results; 3.1. Search results** |
|  | 16b | Cite studies that might appear to meet the inclusion criteria, but which were excluded, and explain why they were excluded. | **3. Results; 3.1. Search results** |
| Study characteristics | 17 | Cite each included study and present its characteristics. | **3. Results; 3.2. Study characteristics** |
| Risk of bias in studies | 18 | Present assessments of risk of bias for each included study. | **3. Results;** **3.3. Risk of bias in included studies** |
| Results of individual studies | 19 | For all outcomes, present, for each study: (a) summary statistics for each group (where appropriate) and (b) an effect estimate and its precision (e.g. confidence/credible interval), ideally using structured tables or plots. | **3. Results; 3.4.** **Effect estimates** |
| Results of syntheses | 20a | For each synthesis, briefly summarise the characteristics and risk of bias among contributing studies. | **3. Results; 3.4. Effect estimates** |
|  | 20b | Present results of all statistical syntheses conducted. If meta-analysis was done, present for each the summary estimate and its precision (e.g. confidence/credible interval) and measures of statistical heterogeneity. If comparing groups, describe the direction of the effect. | **3. Results; 3.4. Effect estimates** |
|  | 20c | Present results of all investigations of possible causes of heterogeneity among study results. | **3. Results; 3.4. Effect estimates** |
|  | 20d | Present results of all sensitivity analyses conducted to assess the robustness of the synthesized results. | **3. Results; 3.5. Sensitivity analysis** |
| Reporting biases | 21 | Present assessments of risk of bias due to missing results (arising from reporting biases) for each synthesis assessed. | **3. Results; 3.6. Publication bias** |
| Certainty of evidence | 22 | Present assessments of certainty (or confidence) in the body of evidence for each outcome assessed. | **3. Results; 3.7. GRADE certainty of evidence** |
| DISCUSSION | | |  |
| Discussion | 23a | Provide a general interpretation of the results in the context of other evidence. | **4. Discussion; 4.1. Summary of main results in this review** |
|  | 23b | Discuss any limitations of the evidence included in the review. | **4. Discussion; 4.4. Strengths and limitations** |
|  | 23c | Discuss any limitations of the review processes used. | **4. Discussion; 4.4. Strengths and limitations** |
|  | 23d | Discuss implications of the results for practice, policy, and future research. | **4. Discussion; 4.3. Implications for future research** |
| OTHER INFORMATION | | |  |
| Registration and protocol | 24a | Provide registration information for the review, including register name and registration number, or state that the review was not registered. | **2. Methods** |
|  | 24b | Indicate where the review protocol can be accessed, or state that a protocol was not prepared. | **2. Methods** |
|  | 24c | Describe and explain any amendments to information provided at registration or in the protocol. | **NA** |
| Support | 25 | Describe sources of financial or non-financial support for the review, and the role of the funders or sponsors in the review. | **Funding** |
| Competing interests | 26 | Declare any competing interests of review authors. | **Conflict of interest** |
| Availability of data, code and other materials | 27 | Report which of the following are publicly available and where they can be found: template data collection forms; data extracted from included studies; data used for all analyses; analytic code; any other materials used in the review. | **Data availability** |

**Supplement 2. The full search strategy for each database**

| Database | Search Strategy |
| --- | --- |
| Pubmed | #1 Myocardial Infarction[Mesh]  #2 Myocardial infarction[Title/Abstract] OR Infarction, Myocardial[Title/Abstract] OR Infarctions, Myocardial[Title/Abstract] OR Myocardial Infarctions[Title/Abstract] OR Heart Attack[Title/Abstract] OR Heart Attacks[Title/Abstract] OR Myocardial Infarct[Title/Abstract] OR Infarct, Myocardial[Title/Abstract] OR Infarcts, Myocardial[Title/Abstract] OR Myocardial Infarcts[Title/Abstract] OR Cardiovascular Stroke[Title/Abstract] OR Cardiovascular Strokes[Title/Abstract] OR Stroke, Cardiovascular[Title/Abstract] OR Strokes, Cardiovascular[Title/Abstract]  #3 #1 OR #2  #4 Heart Failure[Mesh]  #5 Heart Failure[Title/Abstract] OR Cardiac Failure[Title/Abstract] OR Heart Decompensation[Title/Abstract] OR Decompensation, Heart[Title/Abstract] OR Myocardial Failure[Title/Abstract] OR cardiac decompensation[Title/Abstract] OR cardiac insufficiency[Title/Abstract] OR heart insufficiency[Title/Abstract] OR myocardial insufficiency[Title/Abstract]  #6 #4 OR #5  #7 Qili Qiangxin[Title/Abstract] OR Qiliqiangxin[Title/Abstract] OR Qi Li Qiang Xin[Title/Abstract]  #8 randomized controlled trial[pt] OR controlled clinical trial[pt] OR clinical trials as topic[mesh:noexp] OR trial[ti] OR random*[tiab] OR placebo*[tiab]  #9 #3 AND #6 AND #7 AND #8 |
| Cochrane Library | #1 MeSH descriptor: [Myocardial Infarction] explode all trees  #2 (Myocardial infarction OR Infarction, Myocardial OR Infarctions, Myocardial OR Myocardial Infarctions OR Heart Attack OR Heart Attacks OR Myocardial Infarct OR Infarct, Myocardial OR Infarcts, Myocardial OR Myocardial Infarcts OR Cardiovascular Stroke OR Cardiovascular Strokes OR Stroke, Cardiovascular OR Strokes, Cardiovascular):ti,ab,kw  #3 #1 OR #2  #4 MeSH descriptor: [Heart Failure] explode all trees  #5 (Heart Failure OR Cardiac Failure OR Heart Decompensation OR Decompensation, Heart OR Myocardial Failure OR cardiac decompensation OR cardiac insufficiency OR heart insufficiency OR myocardial insufficiency):ti,ab,kw  #6 #4 OR #5  #7 (Qili Qiangxin OR Qiliqiangxin OR Qi Li Qiang Xin):ti,ab,kw  #8 MeSH descriptor: [Randomized Controlled Trial] explode all trees  #9 (controlled clinical trial OR randomized OR random allocation OR clinical trial OR placebo OR drug therapy OR randomly):ti,ab,kw  #10 #8 OR #9  #11 #3 AND #6 AND #7 AND #10 |
| Embase | #1 'heart infarction'/exp  #2 ('Myocardial infarction' OR 'Infarction, Myocardial' OR 'Infarctions, Myocardial' OR 'Myocardial Infarctions' OR 'Heart Attack' OR 'Heart Attacks' OR 'Myocardial Infarct' OR 'Infarct, Myocardial' OR 'Infarcts, Myocardial' OR 'Myocardial Infarcts' OR 'Cardiovascular Stroke' OR 'Cardiovascular Strokes' OR 'Stroke, Cardiovascular' OR 'Strokes, Cardiovascular'):ti,ab,kw  #3 #1 OR #2  #4 'heart failure'/exp  #5 ('Heart Failure' OR 'Cardiac Failure' OR 'Heart Decompensation' OR 'Decompensation, Heart' OR 'Myocardial Failure' OR 'cardiac decompensation' OR 'cardiac insufficiency' OR 'heart insufficiency' OR 'myocardial insufficiency'):ti,ab,kw  #6 #4 OR #5  #7 ('Qili Qiangxin' OR 'Qiliqiangxin' OR 'Qi Li Qiang Xin'):ti,ab,kw  #8 'controlled clinical trial'/exp  #9 (random* OR placebo*):ti,ab  #10 trial:ti  #11 (#8 OR #9 OR #10) AND [embase]/lim  #12 #3 AND #6 AND #7 AND #11 |
| CNKI | (SU=('心肌梗死'+'心肌梗塞'+'心梗') OR TKA=('心肌梗死'+'心肌梗塞'+'心梗')) AND (SU=('心力衰竭' +'心衰'+'心功能不全') OR TKA=('心力衰竭'+'心衰'+'心功能不全')) AND (SU='芪苈强心胶囊' OR TKA='芪苈强心胶囊') AND (FT='随机') |
| Wanfang Data | (主题:(心肌梗死 OR 心肌梗塞 OR 心梗) OR 题名或关键词:(心肌梗死 OR 心肌梗塞 OR 心梗) OR 摘要:(心肌梗死 OR 心肌梗塞 OR 心梗)) AND (主题:(心力衰竭 OR 心衰 OR 心功能不全) OR 题名或关键词:(心力衰竭 OR 心衰 OR 心功能不全) OR 摘要:(心力衰竭 OR 心衰 OR 心功能不全)) AND (主题:芪苈强心胶囊 OR 题名或关键词:芪苈强心胶囊 OR 摘要:芪苈强心胶囊) AND 全部:随机 |
| VIP | (M=(心肌梗死 OR 心肌梗塞 OR 心梗) OR R=(心肌梗死 OR 心肌梗塞 OR 心梗)) AND (M=(心力衰竭 OR 心衰 OR 心功能不全) OR R=(心力衰竭 OR 心衰 OR 心功能不全)) AND (M=芪苈强心胶囊 OR R=芪苈强心胶囊) AND (U=随机) |
| SinoMed | ("心肌梗死"[常用字段:智能] OR "心肌梗塞"[常用字段:智能] OR "心梗"[常用字段:智能]) AND ("心力衰竭"[常用字段:智能] OR "心衰"[常用字段:智能] OR "心功能不全"[常用字段:智能]) AND ("芪苈强心胶囊"[常用字段:智能]) AND ("随机"[全部字段:智能]) |

**Supplement 3. Reports excluded by regarding full texts**

| No. | Excluded studies with different reasons |
| --- | --- |
| 1 | Ineligible participants (n = 10) |
|  | [1] Wei Xiong, Hongzhuan Cai. Effect of rhBNP combined with Qili Qiangxin capsule on cardiac function in patients with acute myocardial infarction complicated with heart failure (rhBNP联合芪苈强心胶囊对急性心肌梗死合并心力衰竭病人心功能的影响). Chinese Journal of Integrative Medicine on Cardio-Cerebrovascular Disease, 2021, 19(6): 967-969.  [2] Weiji Chen. Clinical efficacy of milrinone combined with Qili Qiangxin capsule in the treatment of acute myocardial infarction complicated with heart failure (米力农联合芪苈强心胶囊治疗急性心肌梗死合并心力衰竭的临床疗效). China Practical Medicine, 2021, 16(5): 111-113.  [3] Wenwu Zheng, Li Huang, Qiang Ye, et al. Observation on the efficacy of Qili Qiangxin capsule in patients with cardiac dysfunction after acute myocardial infarction (芪苈强心胶囊对急性心肌梗死后心功能不全患者疗效观察). Journal of Medical Information, 2014(17): 64-64.  [4] Weiyong Zhu. Observation on the efficacy of Qili Qiangxin capsule in patients with acute myocardial infarction and heart failure undergoing interventional therapy (芪苈强心胶囊对行介入治疗的急性心肌梗死并心力衰竭患者疗效观察). China Practical Medicine, 2015, 10(35): 170-171.  [5] Jinyu Song, Xin Du, Jihong Shi, et al. Effect of Qili Qiangxin capsule combined with milrinone on arterial blood flow and cardiac function in patients with myocardial infarction complicated with heart failure (芪苈强心胶囊联合重组人脑利钠肽治疗心肌梗死合并心力衰竭的效果观察). Shaanxi Journal of Traditional Chinese Medicine, 2020, 41(4): 493-495.  [6] Xuannian Chen, Runjun Li. Observation on the effect of Qili Qiangxin capsule combined with recombinant human brain natriuretic peptide in the treatment of myocardial infarction complicated with heart failure (芪苈强心胶囊联合重组人脑利钠肽治疗心肌梗死合并心力衰竭的效果观察). Chinese Community Doctors, 2024, 40(3): 94-96.  [7] Yanke Pan, Chuang Hui. Effect of Qili Qiangxin capsule combined with levosimendan in the treatment of patients with acute myocardial infarction complicated with heart failure (芪苈强心胶囊联合左西孟旦治疗急性心肌梗死合并心力衰竭患者的效果). Medical Journal of Chinese People's Health, 2024, 36(1): 96-98, 102.  [8] Jingzhai Fu, Nan Huang, Chunling Liu, et al. Observation on the efficacy of Qili Qiangxin capsule in the treatment of 32 cases of acute myocardial infarction complicated with heart failure (芪苈强心胶囊治疗急性心肌梗死合并心力衰竭32例疗效观察). Hebei Journal of Traditional Chinese Medicine, 2010, 32(1): 93-95.  [9] Shuren Dai, Yafei Li, Yuhe Zhao, et al. Analysis of the effect of Qili Qiangxin capsule in the treatment of elderly patients with acute myocardial infarction complicated with heart failure (芪苈强心胶囊治疗老年人急性心肌梗死合并心力衰竭的效果分析). Shenzhen Journal of Integrated Traditional Chinese and Western Medicine, 2022, 32(14): 70-73.  [10] Ning Zhang, Huipeng Wu, Bin An. Clinical value of recombinant human brain natriuretic peptide combined with Qili Qiangxin capsule in the treatment of elderly patients with acute myocardial infarction complicated with heart failure (重组人脑利钠肽联合芪苈强心胶囊治疗老年急性心肌梗死合并心力衰竭的临床价值). Chinese Journal of Geriatric Heart Brain and Vessel Diseases, 2022, 24(7): 685-688. |
| 2 | Ineligible interventions (n = 1) |
|  | [1] Wanfu Cui, Kunmei Han. Efficacy observation of Qili Qiangxin capsule as adjunctive treatment for heart failure after myocardial infarction (芪苈强心胶囊辅治心肌梗死后心力衰竭疗效观察). Heilongjiang Medicine and Pharmacy, 2012, 35(1): 47. |
| 3 | The duration of treatment was inconsistent between the treatment and control groups (n = 1) |
|  | [1] Xin Xu, Lanlan Wang. Efficacy of Qili Qiangxin capsule combined with rhBNP in patients with heart failure after acute myocardial infarction (芪苈强心胶囊联合rhBNP对急性心肌梗死后心力衰竭患者的疗效). International Medicine and Health Guidance News, 2024, 30(8): 1269-1273. |

**Supplement 4. Detailed background Western medicine treatments in the included RCTs**

| Study | Background Western medicine treatment | Between-group comparability |
| --- | --- | --- |
| Bai LQ2013 | Aspirin enteric-coated tablets 100 mg once daily; clopidogrel 75 mg once daily; rosuvastatin 10 mg once daily at night; benazepril 2.5–10 mg once daily, or irbesartan 75–300 mg once daily if intolerant; bisoprolol 1.25–5 mg once daily; trimetazidine 20 mg three times daily; digoxin 0.125 mg once daily; furosemide 20 mg twice daily; spironolactone 20 mg twice daily; sodium nitroprusside 50 mg in 50 mL 5% glucose infusion at 3–15 mL/h; dopamine 180 mg in 32 mL normal saline at 5 mL/h; dobutamine according to clinical condition. | Same Western medicine treatments in both groups; QLQX was added only in the intervention group. |
| Chen SH2019 | Furosemide; digoxin; enalapril. Specific doses and frequencies were not reported. | Same Western medicine treatments in both groups; QLQX was added only in the intervention group. |
| Chen YQ2020 | Conventional treatment according to the 2014 Chinese guideline. Specific drugs, doses and frequencies were not reported. | Same Western medicine treatments in both groups; QLQX was added only in the intervention group. |
| Chen YZ2025 | Aspirin 100 mg once daily; clopidogrel 75 mg once daily; atorvastatin 20 mg once daily; metoprolol succinate sustained-release 47.5 mg once daily; dapagliflozin 10 mg once daily; sacubitril/valsartan 50 mg twice daily | Same Western medicine treatments in both groups; QLQX was added only in the intervention group. |
| Han L2024 | β-blockers, vasodilators, antiplatelet agents, lipid-stabilising therapy and diuretics; sacubitril/valsartan initially 50 mg twice daily, increased every 2–4 weeks according to clinical condition up to 200 mg per dose. | Same Western medicine treatments in both groups; QLQX was added only in the intervention group. |
| Hou XL2010 | ACEI or ARBs, β-blockers and diuretics; in severe cases, dopamine with sodium nitroprusside or nitroglycerin, adjusted according to blood pressure and clinical condition. Specific doses were not reported. | Same Western medicine treatments in both groups; QLQX was added only in the intervention group. |
| Liu YJ2012 | Digoxin (or intravenous cedilanid followed by oral digoxin maintenance), furosemide, spironolactone, ACEI and lipid-lowering drugs; nitroglycerin or sodium nitroprusside by infusion in severe cases. Specific doses and frequencies were not reported. | Same Western medicine treatments in both groups; QLQX was added only in the intervention group. |
| Liu Y2023 | Antiplatelet therapy, lipid-lowering therapy, diuretics, ventricular rate control, anti-infective therapy and oxygen therapy; levosimendan 12.5 mg in 45 mL 5% glucose at 1.5 mL/h by infusion, once weekly for 3 weeks, with adjustment according to blood pressure. | Same Western medicine treatments in both groups; QLQX was added only in the intervention group. |
| Luo F2019 | Antiplatelet agents, anticoagulants, lipid-lowering therapy, β-blockers, ACEI, vasodilators and diuretics. Specific drugs, doses and frequencies were not reported. | Same Western medicine treatments in both groups; QLQX was added only in the intervention group. |
| Pang J2014 | Standardised pharmacological treatment for AMI; additional treatment according to clinical condition included diuretics, ACEI or ARBs, β-blockers, positive inotropic agents and mineralocorticoid receptor antagonists. Specific drugs, doses and frequencies were not reported. | Same Western medicine treatments in both groups; QLQX was added only in the intervention group. |
| Qi MJ2024 | Lipid-lowering therapy, antithrombotic therapy, diuretics and positive inotropic therapy; dapagliflozin initially 5 mg once daily, increased to 10 mg once daily according to clinical condition and tolerance. | Same Western medicine treatments in both groups; QLQX was added only in the intervention group. |
| Qiao DF2018 | ACEI, β-blockers, diuretics and vasodilators. Specific drugs, doses and frequencies were not reported. |  |
| Shi XL2024 | Anticoagulant and thrombolytic therapy; recombinant human brain natriuretic peptide (rhBNP), loading dose 1.5 μg/kg followed by continuous intravenous infusion at 0.0075 μg/(kg·min), adjusted to 0.0075–0.02 μg/(kg·min) according to clinical condition for 72 h; empagliflozin 10 mg once daily. | Same Western medicine treatments in both groups; QLQX was added only in the intervention group. |
| Wang ZJ2015 | ACEI/ARB, β-blocker, spironolactone, atorvastatin, and digoxin, with drugs administered to the target or maximum tolerated doses. Specific doses and frequencies were not reported. | Same Western medicine treatments in both groups; QLQX was added only in the intervention group. |
| Xi K2011 | Digoxin, diuretics, ACEI/ARB or β-blocker (at the maximum target dose), and atorvastatin. Specific doses and frequencies were not reported. | Same Western medicine treatments in both groups; QLQX was added only in the intervention group. |
| Xie ZQ2022 | Anticoagulant therapy, antiplatelet therapy, glucose/lipid metabolism-regulating therapy and diuretics; sacubitril/valsartan initially 50 mg twice daily, gradually increased to 200 mg twice daily according to tolerance. | Same Western medicine treatments in both groups; QLQX was added only in the intervention group. |
| Note: ACEI, angiotensin-converting enzyme inhibitor; AMI, acute myocardial infarction; ARB, angiotensin II receptor blocker; β-blocker, beta-adrenergic receptor blocker; QLQX, Qili Qiangxin Capsule; rhBNP, recombinant human brain natriuretic peptide | | |

**Supplement 5. Subgroup analysis**

**N-terminal pro-B-type natriuretic peptide (NT-proBNP)**

**Supplemental Figure 1.** Forest plot comparing QLQX plus WM to WM alone on NT-proBNP.

**Left ventricular ejection fraction (LVEF)**

**Supplemental Figure 2.** Forest plot comparing QLQX plus WM to WM alone on LVEF.

**Left ventricular end-systolic diameter (LVESD)**

**Supplemental Figure 3.** Forest plot comparing QLQX plus WM to WM alone on LVESD.

**Left ventricular end-diastolic diameter (LVEDD)**

**Supplemental Figure 4.** Forest plot comparing QLQX plus WM to WM alone on LVEDD.

**High-sensitivity C-reactive protein (hs-CRP)**

**Supplemental Figure 5.** Forest plot comparing QLQX plus WM to WM alone on hs-CRP.

Abbreviations: QLQX, Qili Qiangxin Capsule; WM, Western medicine; w, week; m, month
